# Impact of Basketball Match on the Pre-Competitive Anxiety and HRV of Youth Female Players

**DOI:** 10.3390/ijerph19137894

**Published:** 2022-06-27

**Authors:** Juan M. García-Ceberino, Juan Pedro Fuentes-García, Santos Villafaina

**Affiliations:** 1 Faculty of Humanities and Social Sciences, University of Isabel I, 09003 Burgos, Spain; juanmanuel.garcia.ceberino@ui1.es; 2 Optimization of Training and Sports Performance Research Group (GOERD), University of Extremadura, 10003 Cáceres, Spain; 3 Universidad de Extremadura, Facultad de Ciencias del Deporte, Av. de la Universidad S/N, 10003 Cáceres, Spain; svillafaina@unex.es; 4 Departamento de Desporto e Saúde, Escola de Saúde e Desenvolvimento Humano, Universidade de Évora, 7004-516 Évora, Portugal

**Keywords:** autonomic modulation, anxiety, physical activity, sport

## Abstract

The present study aimed to investigate the impact of a basketball competition on the pre-competitive anxiety and Heart Rate Variability (HRV) of young female basketball players. A total of 12 female basketball players participated in this cross-sectional study. Girls had a mean age of 14 (1.41) years old and a mean experience of 4 (0.85) years practicing basketball. The pre-competitive anxiety and the HRV was assessed the week before and immediately before and after the match. Results showed a significant reduction (*p*-value < 0.05) of the several HRV variables after the match compared to baseline and pre-competition measures. However, differences between baseline and pre-competition were not found. Furthermore, a significant increase in somatic anxiety after the competition was reported. Taking into account HRV, somatic anxiety, and cognitive anxiety results, young female basketball players did not exhibit an increase in pre-competition anxiety. HRV measurements before competition can help coaches and physical trainers to identify female players with higher pre-competitive anxiety and propose intervention to manage it. Future studies should investigate the impact of coaches and parents on the pre-competitive anxiety of female and male basketball players.

## 1. Introduction

The Heart Rate Variability (HRV), which measures successive heartbeat variation over an interval of time [1], is considered a non-invasive method that provides information regarding the balance between sympathetic and parasympathetic nervous systems. When the sympathetic nervous system has a predominant activity, HRV is reduced and more regular. Therefore, it has been related to the regulatory capacity of the organism to adapt to different situations or environments [2]. This is why HRV is considered a biomarker of overtraining and an indicator of stress [3]. Previous studies have employed the HRV to explore the pre-competitive anxiety in sports such as soccer [4], tennis [5], swimming [6,7], mountain bike cycling [8], or BMX cycling [9]. Results showed that HRV tended to be decreased due to the anxiogenic effect of the competition [4,8,9].

Anxiety is a complex response that mixes the somatic dimensions (indicators of heart rate, muscle tension, or respiratory rate could be identified as indicators) and the cognitive (where self-evaluation, expectations, or concentration have a crucial role) [10]. This state could develop adverse effects, feelings of apprehension, and tension associated with a high level of activation of the organism [10]. On the sports field, athletes suffer from anxiety because they are pushed to use all their available resources to achieve the maximum performance [11]. Thus, previous studies have identified pre-competitive anxiety induced by competition [4,12]. Therefore, anxiety, specifically pre-competitive anxiety, is one of the most studied emotional responses to competitive sports participation in young athletes [11]. In this regard, gender differences can be observed in levels of anxiety [13,14,15]. Previous studies have found that girls exhibited greater levels of pre-competitive anxiety [16,17,18]. Therefore, the study of pre-competitive anxiety in female athletes using behavioral and physiological measures may help improve knowledge on this relevant topic. However, the role of coaches and parents could influence the anxiogenic response of young athletes. In this regard, parents and coaches focused on winning could decrease the enjoyment as well as increase the anxiety of young athletes [19].

Regarding pre-competitive anxiety in basketball, the majority of the studies conducted on this topic have focused on how pre-competitive anxiety could influence or be influenced by other variables such as performance [20], the number of minutes [21], sex [16], playing at home or away [22], parental attitudes [23], or the level of the rival [24]. However, no studies have compared pre-competitive anxiety in different moments such as baseline, pre-competition, and post-competition. Nevertheless, Arruda et al. [25] explored the effect of playing matches against different opponent teams on pre-match testosterone concentration, pre-to-post match cortisol concentration, and pre-competitive anxiety in 20 elite male basketball players. Hoover et al. [26] investigated pre-competitive anxiety at baseline and prior to non-conference, conference, and state tournament games. Furthermore, Mohamed Nasr [27] showed that young female basketball players exhibited higher levels of anxiety than young male players prior to a competition. Therefore, there is a need for studies that analyze the pre-competitive anxiety in young players, specifically in women, where previous studies have reported higher values of pre-competitive anxiety [16]. Therefore, the aim of the present study was to investigate the impact of a basketball competition on the pre-competitive HRV of young female basketball players. We hypothesized, based on previous research [4,12], that (1) HRV would be decreased and pre-competitive anxiety would be increased before a competition compared to a baseline; and (2) post-competition HRV would be higher than that obtained in the baseline and pre-competition measurements.

## 2. Materials and Methods

### 2.1. Participants

The G*Power software 3.1.9.4 (Kiel University, Kiel, Germany) was employed to estimate that a sample size of 10 achieves 80% power to detect significant differences with an alpha of 0.005 using the Wilcoxon signed-rank test. Data from young female basketball players [27] on the pre- and post-competitive anxiety were used to make the calculation (23.13 (7.12) for pre-competitive anxiety and 18.80 (3.89) for post-competitive anxiety with a correlation index of 0.8). Since three evaluations were required to conduct the protocol, we recruited 20% more participants to ensure the minimum participation. Thus, a cohort of 12 young female basketball players from a public institution participated in this cross-sectional study. Girls had a mean age of 14.00 (1.41) years old and a mean experience of 4.00 (0.85) years practicing basketball. Participants weighed 57.18 (5.86) kg with heights of 1.67 (0.05) m.

Participants belonged to a basketball team that finished the proximity league at second position (11 wins and 3 losses). Only teams close to the female players’ locality participated in this league.

After that, this second position allowed them to qualify to play in the gold playoffs that gave access to the Spanish cadet championship. Only the champion team of these playoffs would play the championship. Specifically, the analyzed match corresponded to the quarter-finals match. They lost this match, and therefore this was the last match of the season. The proximity league and playoffs took place in southwest Spain in March 2022.

Aims of the study and procedures were explained to girls and parents prior to voluntary participation. Then, all participants, parents, or legal guardians agreed and provided written consent to participate in this cross-sectional study. Procedures were approved by the university research ethics committee (approval number: 180/2019). To guarantee the ethical considerations for scientific investigations with human beings, the study was conducted according to the ethical guidelines of the Declaration of Helsinki of 1975 (modified in subsequent years), and Organic Law 3/2018 of 5 December on the protection of personal data and guarantee of digital rights (BOE, 294, 6 December 2018).

### 2.2. Variables and Instruments

The Spanish version of the Competitive State Anxiety Inventory–2R (CSAI-2R) was employed to measure the somatic anxiety, cognitive anxiety, and self-confidence [28,29]. This questionnaire has 17 items (in a 4-point Likert scale) that ranged from “not at all” to “very much so”. The somatic anxiety subscale has 7 items, with a minimum score of 7 and a maximum score of 28. Cognitive anxiety was calculated using 5 items, ranging the overall score from 5 to 20 points. The self-confidence subscale was calculated using 5 items, where scores ranged from 5 to 20 points.

Four Polar RS800CX (Finland) heart rate monitors [30] were employed to evaluate the HRV. The Task Force of the European Society of Cardiology and the North American Society of Pacing and Electrophysiology [31] and Catai et al. [32] recommendations were followed to record the HRV. A five minute short-term record at sitting position was conducted in three moments: baseline, before, and after the match.

HRV data was analyzed using the Kubios HRV software (v. 3.3) [33]. The threshold-based beat correction algorithm was used for handling artifacts. This algorithm compares every RR interval value against a local average interval. The median filtering the RR interval time series was used to obtain the local average. This means that single outliers in RR interval time series did not affect the local average. If an RR interval differs from the local average more than 0.25 s, the interval is identified as an artifact [34]. Artifacts were replaced using spline interpolation. Slow nonstationary trends were removed using the smoothness prior method with a Lambda value of 500 [35].

Time, frequency, and non-linear measures were extracted using Kubios HRV software. In the time domain, different measures were included: mean heart rate (mean HR), RR intervals (time between intervals R-R), RR50 count divided by the total number of all RR ranges (Pnn50), the standard deviation of all normal-to-normal RR intervals (SDNN), and the square root of differences between adjacent RR intervals (RMSSD). In the frequency domain, the following measures were included: low frequency (LF, 0.04–0.15 Hz) and high frequency (HF, 0.15–0.4 Hz) ratio (LF/HF) and Total Power. Lastly, in the non-linear measures, the following variables were included: RR variability from heartbeat to short term Poincaré graph (width) (SD1), RR variability from heartbeat to long-term Poincaré graph (length) (SD2), and Sample Entropy (SampEn).

Higher values of SDNN, RMSSD, SD1, and SampEn, are associated with parasympathetic modulation, and a reduction in these previous indexes or lower values of LF/HF and SD2 are associated with an increase in sympathetic modulation [36,37,38,39]. Nevertheless, due to complex sympathetic–parasympathetic interactions, the underlying mechanisms of frequency-domain and non-linear measures are still less well established. Thus, controversial results can be found in the literature [40].

### 2.3. Procedure

Catai, Pastre, de Godoy, da Silva, de Medeiros Takahashi and Vanderlei [32] recommendations for assessing and reporting HRV data were followed. A short-term record (5 min at sitting position) was conducted at baseline, pre-competition, and post-competition. To avoid interactions between players, a researcher was present so that the room was calm, and players were at their places (sitting position), encouraged to remain silent. The room temperature where measurements took place ranged between 21.7 °C to 22.5 °C, and the humidity ranged between 43.1% and 46.8%.

**Figure 1:**
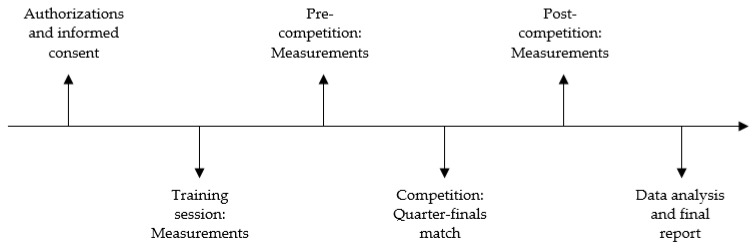
Timeline of the study procedure.

Figure 1 depicts the study procedure timeline. The HRV measurements were on the same day of the week and at the same time of the match and training session. All measurements were performed in the same changing room. Players were familiarized with the heart rate monitors, procedures, and environment. In addition, participants were encouraged not to take any drugs, drinks, or other substances that could affect the nervous system 24 h before undergoing the protocol.

The same following procedures were carried out:

- Baseline: HRV data and pre-competitive anxiety were evaluated three days before the quarter-finals match. All participants were evaluated before the warm-up in the same training session.
- Pre-competition: HRV and pre-competitive anxiety were collected five minutes before starting the quarter-finals match’s warm-up.
- Post-competition: HRV and pre-competitive anxiety were collected immediately after the players arrived at the changing room.

### 2.4. Statistical Analysis

The Statistical Package for Social Sciences, version 25 (SPSS) was employed to analyze the data. The significance level was *p*-value < 0.05. According to the Shapiro–Wilk test, non-parametric analyses were performed.

A Friedman test was conducted to explore differences between baseline, pre-competition, and post-competition measurements. Then, a Wilcoxon signed-rank test was employed to conduct pairwise comparisons. Bonferroni corrections for multiple comparisons were applied. In addition, Kendall—W effect sizes [r] were calculated and classified as: < 0.1 as a small effect, between 0.1 and 0.5 a medium effect, and > 0.5 as a large effect [41,42].

## 3. Results

Participants´ characteristics are depicted in Table 1. Young female basketball players had a mean age of 14.00 (1.41) years, a mean body mass index of 20.60 (1.78) kg/m^2,^ and basketball experience of 4.00 (0.85).

Table 2 shows the HRV values at baseline, pre-competition, and post-competition. Friedman’s test revealed significant differences in all the variables studied in the time, frequency, and non-linear measures. Pairwise comparison showed that HRV significantly decreased post-competition compared with baseline and pre-competition values (mean HR, RR-interval, RMSSD, and SD1). In the same line, pNN50, SDNN, HFnu, total power, and SD2 showed a significant reduction after a competition compared with the pre-competition. Furthermore, LFnu and LF/HF showed a significant increase post-competition when compared with pre-competition. Moreover, significant differences were found between baseline and post-competition in the SampEn variable. Differences between baseline and pre-competition were not found in any of the variables (see Table 2 for further details).

**Figure 2:**
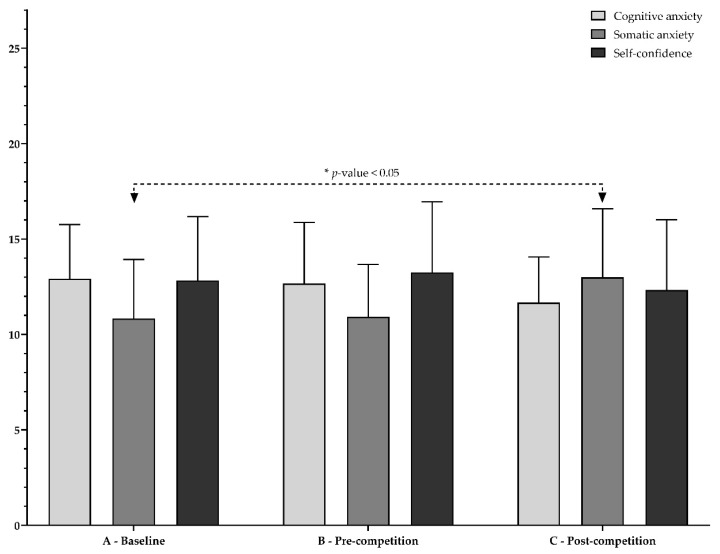
Baseline, pre-competition, and post-competition values of cognitive anxiety, somatic anxiety, and self-confidence. Note: * A < C.

**Figure 3:**
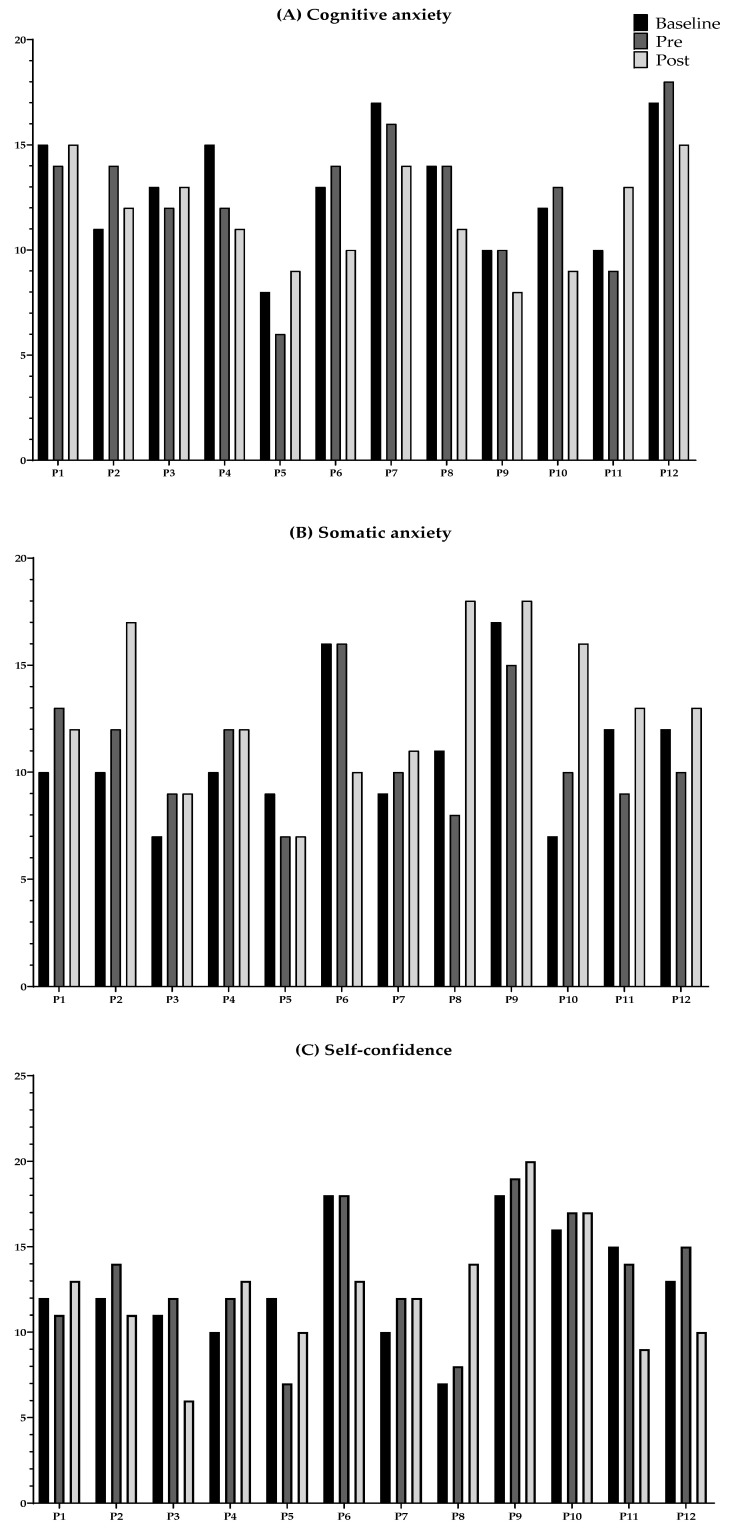
Individual evolution of cognitive anxiety (**A**), somatic anxiety (**B**), and self-confidence (**C**) at baseline, pre- and post-competition in the 12 young female basketball players.

Figure 2 and Figure 3 show the cognitive anxiety, somatic anxiety, and self-confidence at baseline, pre-competition, and post-competition. As shown, a significant increase in somatic anxiety (*p*-value < 0.05) was found between baseline and post-competition in young female basketball players (see Figure 2). Figure 3 shows the individual evolution of somatic anxiety (panel A), cognitive anxiety (panel B), and self-confidence (panel C) for each participant.

## 4. Discussion

Pre-competition and post-competition anxiety can negatively affect the enjoyment and performance of young basketball players. This study aimed to investigate the impact of a basketball competition on the pre-competitive and post-competitive HRV, cognitive anxiety, somatic anxiety, and self-confidence of young female basketball players. Results showed a significant reduction of the several HRV variables after the match compared to baseline and pre-competition measures. However, differences between baseline and pre-competition were not found. Furthermore, a significant increase in somatic anxiety after the competition was reported. Taking into account HRV, somatic anxiety, and cognitive anxiety results, young female basketball players did not exhibit an increase in pre-competition anxiety.

Usually, sport competition demands athletes give all their efforts which can be perceived as stressful [43,44]. In the same line, previous studies have found that competition can increase the pre-competitive anxiety of athletes [4,8,9]. Regarding young athletes, a previous study focused on youth-associated male and female tennis players showed a significant reduction in the HRV [5] to pre-competitive anxiety. However, significant differences between pre and post values of cognitive anxiety, somatic anxiety, and self-confidence were not found in young tennis players [17]. Regarding basketball, previous studies focused on young athletes showed contradictory results. Mohamed Nasr [27] showed that pre-competitive anxiety was significantly higher before than after a basketball match. However, Ortega Vila et al. [45] showed low levels of competitive anxiety in all three subscales (somatic anxiety, worry, and concentration/disruption). In the same line, our results, in addition to not showing significant pre-competition anxiety, participants exhibited lower values of somatic anxiety and cognitive anxiety. These results are also supported by data obtained from HRV analyses. However, our results also showed a significant increase in somatic anxiety after the match. The match consisted of four quarters of ten minutes. Furthermore, considering the actions performed by basketball players (sprints, jumps, change of directions, shots, or blocking) [46] and the intermittent nature of the game [47], the somatic symptoms that players perceived could also be derived from the fatigue caused by the match. However, we cannot discard that it was a psychological effect of the match since the participants lost it.

The anxiogenic response to competition in young athletes seems to be modulated by factors such as genre, experience, sport modality, or even parents or coach conduct. In this regard, previous studies have reported that female athletes showed higher pre-competitive anxiety than male athletes [13,48,49,50,51]. This could be due to an increase in somatic symptoms and a decline of self-confidence before competition [13,48,49,50,51]. However, in our study, we did not find a significant increase in pre-competitive anxiety. In this regard, team sports athletes showed less pre-competitive anxiety than single-sport athletes [52]. This can be due to the responsibility of success or failure not falling solely on one athlete [53]. This may partially explain why in our study we did not find pre-competition anxiety in young female basketball players. Another relevant factor is the role of coaches and families. A previous study showed that parents and coaches who were focused on performance could increase the anxiety of young athletes [19]. In addition, the presence of parents during competition could increase the pre-competition anxiety of basketball players [23]. Thus, a previous study developed an intervention to reduce the competitive pressure on athletes to parents and coaches with significant reduction of pre-competitive anxiety in boys and girls participating in community-based basketball programs [54]. In the same line, Ortega Vila, Robles Rodriguez, Gimenez Fuentes-Guerra, Franco Martin, Jimenez Sanchez, Duran Gonzalez, and Abad Robles [45] showed that in the Real Madrid Foundation, where victory is not presented as the main goal, youth basketball players did not exhibit pre-competition anxiety. Therefore, the results can be derived from an adequate treatment of the competition by the parents and the team’s coach.

The HRV, apart from giving us information regarding the pre-competition anxiety level of athletes [4], has been used to manage fatigue in soccer [55,56], paddle [57], or basketball [58] to avoid overtraining [59]. In this regard, our results follow the same tendency obtained in a previous study after a paddle game [57]. These same authors observed a reduction of the HRV after a paddle game compared to a baseline. As in our study, they showed changes in the HRV that can be interpreted as a decrease in the parasympathetic modulation [60]. In addition, our study also reinforces the hypothesis of RMSSD as a biomarker for detecting fatigue [61,62]. This is relevant since HRV values cannot be voluntarily altered as questionnaires can be. Thus, HRV can be used by coaches and physical trainers as an objective tool to detect fatigue or even pre-competitive anxiety states [3].

The federation that used to organize the proximity league and the playoffs, in which the young female basketball players played, has been organizing the “Basketball and Women’s Cup” in recent years. This cup was created as a consequence of the girls’ abandonment of basketball after playing in the cadet category which has been occurring regularly in the region. In this regard, the levels of pre-competitive anxiety obtained in our study could be associated with a greater enjoyment of playing basketball and competition and a greater intention to continue playing this sport at higher categories (youth and senior category). Moreover, the fact of training basketball in a public institution favors the results obtained. As has been shown [63], coaches think that parents whose children play sports in public institutions are more interested in their children’s health and sports training than in performance. In the same line, coaches think that clubs prioritize the competition and performance.

The present study has some limitations that should be acknowledged. First, the relatively small sample size (N = 12) could mean that only great differences had achieved the significance level. However, to the best of our knowledge, this is the first study exploring the effects pre- and post-competition of a basketball competition on young female players. In addition, in future studies, the players’ perceptions of coaches and parents would help clarify the role of these behaviors on the psychological and physiological variables of athletes. Furthermore, future studies should compare young female and male players to investigate differences in pre-competition anxiety. Second, the trait anxiety of athletes was not registered. However, a previous study found significant correlation between trait anxiety and pre-competitive anxiety in young tennis players [17]. Furthermore, future studies are encouraged to ask the athletes directly about their opinion on the match. This qualitative data would provide interesting data regarding the individual perception about pre-competitive anxiety.

As a practical implication of the present study, the use of HRV as a tool to manage pre-competitive anxiety clearly emerges. As commented above, this physiological measure cannot be voluntarily altered and can be complemented by the behavioral data [3]. Thus, coaches and physical trainers should include pre-competitive HRV to observe the anxiogenic response of their athletes. The multi-faceted nature of anxiety in young athletes makes it necessary to implement adequate strategies on- and off-court according to the specific needs of their players. Therefore, increasing the knowledge regarding the anxiogenic response of athletes would be necessary to reduce and manage pre-competitive anxiety, improving the effectiveness of interventions focused on reducing pre-competitive anxiety [64]. Specifically, these interventions should be focused on girls due to the high levels that they presented [16,17,18]. In addition, due to the influence of parents and coaches on athletes’ anxiety responses, institutions and federations are encouraged to incorporate this aspect into the formative process of coaches. Moreover, at the beginning of the season, parents should be trained in the three factors: competition attitude, communication, and environment [63]. This training will generate a positive coach–parent relationship, increasing the sports adherence of girls.

## 5. Conclusions

Pre-competitive anxiety was not identified in young female basketball players. HRV, cognitive anxiety, and somatic anxiety variables did not significantly change between baseline and pre-match measurements. However, HRV decreased after the match compared to baseline and pre-assessments. In addition, somatic anxiety increased after the match. Thus, this treatment of the competition, and based on the results obtained, may be a predictor for young female basketball players to enjoy competition and not feel anxiety; therefore, the possibility of abandoning the practice of this sport will be lower.

## Figures and Tables

**Table 1:** Descriptive data of participants.

Variables	Mean (SD)	95% CI
Age (years)	14.00 (1.41)	13.10–14.90
Basketball experience (years)	4.00 (0.85)	3.46–4.54
Height (m)	1.67 (0.05)	1.63–1.70
Weight (kg)	57.18 (5.86)	53.45–60.90
Body Mass Index (kg/m^2^)	20.60 (1.78)	19.46–21.73

Note: SD = standard deviation; m = meters; kg = kilograms; CI = confidence interval.

**Table 2:** Differences in the HRV at baseline, pre-competition, and post-competition of young female basketball players.

Variables	Baseline Mean (SD)	Pre-Competition Mean (SD)	Post-Competition Mean (SD)	*p*-Value	Effect Size	Pairwise Comparisons
mean HR	85.33 (10.17)	89.91 (11.34)	104.30 (11.17)	<0.001	2.58	A > C; B > C
RR-interval	718.48 (85.75)	683.58 (91.57)	683.58 (586.91)	0.001	2.53	A > C; B > C
pNN50	18.77 (20.07)	17.34 (14.61)	5.72 (8.70)	0.001	2.53	B > C
SDNN	42.55 (18.15)	44.87 (14.66)	31.18 (13.41)	0.009	1.58	B > C
RMSSD	41.23 (28.04)	37.00 (17.01)	21.82 (13.91)	<0.001	2.58	A > C; B > C
HFnu	32.75 (16.50)	33.29 (12.92)	23.89 (7.93)	0.028	1.19	B > C
LFnu	67.07 (16.53)	66.65 (12.93)	76.06 (7.95)	0.028	1.19	C > B
LF/HF	2.89 (1.99)	2.67 (2.22)	4.36 (4.45)	0.028	1.19	C > B
Total power	1729 (1470)	1874 (1027)	1095 (1294)	0.050	1	B > C
SD1	29.19 (19.86)	26.91 (12.06)	15.45 (9.85)	<0.001	2.58	A > C; B > C
SD2	51.87 (18.38)	57.02 (17.32)	41.10 (16.62)	0.028	1.19	B > C
SampEn	1.64 (0.35)	1.44 (0.28)	1.07 (0.33)	0.004	1.86	A > C

Note: A: baseline; B: pre-competition; C: post-competition. HR = heart rate; RR = time between intervals R-R; pNN50 = percentage of intervals >50 ms different from the previous interval; SDNN = the standard deviation of all normal to normal RR intervals; RMSSD = the square root of the mean of the squares of the successive differences of the interval RR; HFnu = high frequency; LFnu = low frequency; LF/HF = low frequency (LF) ratio (ms^2^) / high frequency (HF) ratio (ms^2^); total power = the sum of all the spectra; SD1 = dispersion, standard deviation, of points perpendicular to the axis of line-of-identity in the Poincaré plot; SD2 = dispersion, standard deviation, of points along the axis of line-of-identity in the Poincaré plot; SampEn = Sample Entropy.

## Data Availability

Data will be available upon reasonable request to the corresponding author.
